# Papular purpuric gloves and socks syndrome in a patient with previous SARS-CoV-2 infection

**DOI:** 10.1016/j.jdcr.2021.08.019

**Published:** 2021-08-25

**Authors:** Partik Singh, Sylvana Brickley, Kathleen Mannava, Francisco Tausk

**Affiliations:** Department of Dermatology, University of Rochester Medical Center, Rochester, New York

**Keywords:** COVID, COVID-19, infections, infectious disease, inflammatory, viral exanthema

## Introduction

Papular purpuric gloves and socks syndrome is a pruritic dermatosis that is typically associated with parvovirus B19 and other viral infections.^1^ We present a case of a patient with papular purpuric gloves and socks syndrome who was recently diagnosed with COVID-19. While numerous cutaneous manifestations of COVID-19 infection have been reported thus far,^2^ to our knowledge, this dermatosis has not been reported.

## Case report

A 62-year-old woman presented to the emergency department reporting several days of a pruritic rash that started on the dorsal aspects of the hands and then appeared on the dorsal aspects of the feet. She was afebrile with otherwise unremarkable vital signs and was discharged on triamcinolone 0.1% cream twice daily. Two days later, the rash had not improved, so she called her primary care physician and was prescribed fluocinonide 0.05% ointment twice daily over the phone. The next day, her pruritus improved, but her rash continued to spread to include her left thigh, which led the patient to seek further evaluation from a dermatologist.

When the patient was evaluated for the first time in the dermatology clinic, approximately 10 days after the rash first appeared, the rash had not resolved despite topical treatment. Further interview revealed that she had tested positive for SARS-CoV-2 infection as demonstrated by positive polymerase chain reaction 6 weeks before the rash appeared. She reported that she had recovered from COVID-19 at home without the need for supportive care.

**Fig 1:**
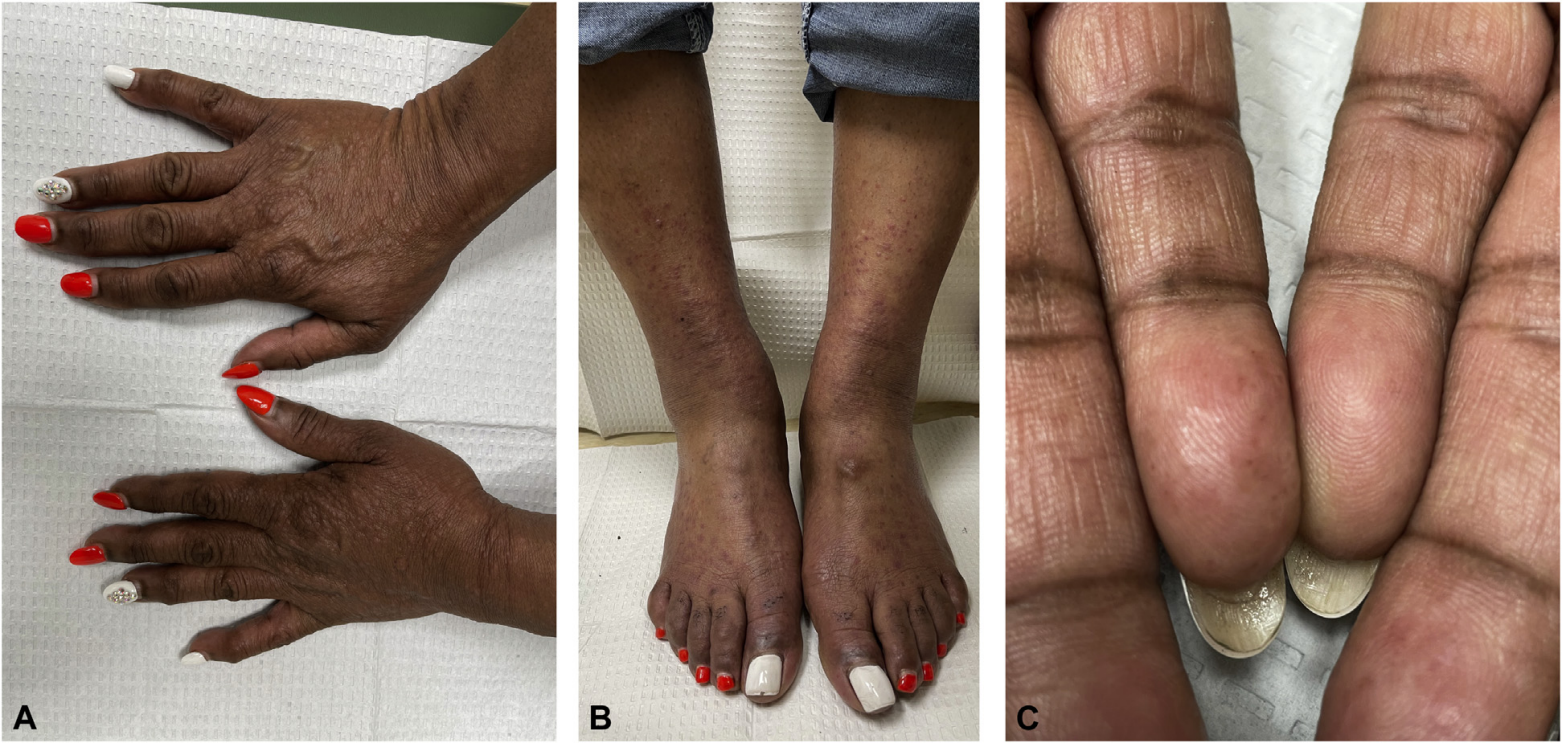
Papular purpuric gloves and socks-distributed eruption. Clinical images on presentation demonstrated violaceous papules coalescing into plaques on the dorsal aspects of the (**A**) hands and (**B**) feet. **C**, Some petechiae were noted on the palmar surface of the fingertips.

**Fig 2:**
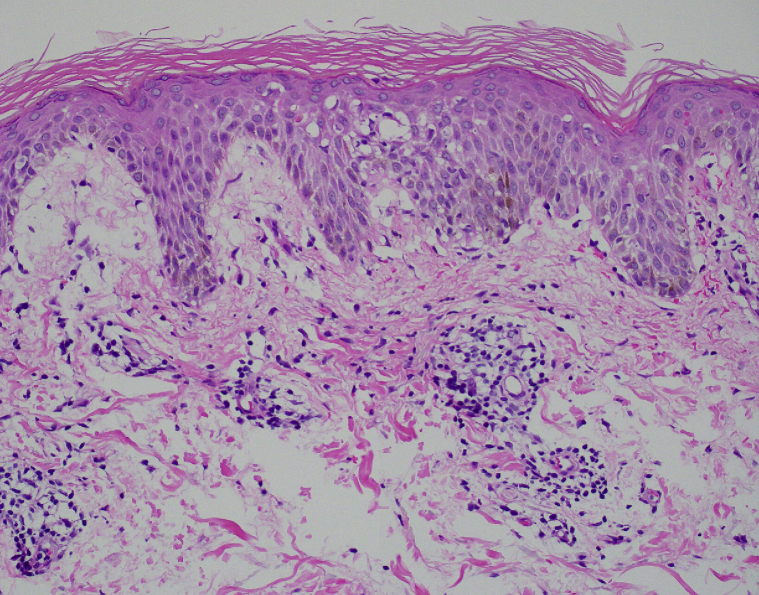
Histopathologic features. Hematoxylin-eosin–stained sections showed a thin epidermis with focal spongiosis and papillary dermal edema. Extravasated erythrocytes and a lymphocytic perivascular infiltrate were noted in the superficial dermis. In the appropriate clinical context, these findings would support a diagnosis of papular purpuric gloves and socks syndrome.

On examination, she had unremarkable vital signs, Fitzpatrick V skin type, and violaceous smooth papules coalescing into plaques on the dorsal aspects of the hands and feet and to a lesser extent on the anterior aspect of the left thigh (Fig 1). Petechial macules on the fingertips and shins were also noted. There was no involvement of the mucosa, palms, or soles. A 3-mm punch biopsy obtained from a representative lesion on the left dorsal hand demonstrated focal epidermal spongiosis, papillary dermal edema with extravasated erythrocytes, and a lymphocytic perivascular infiltrate in the superficial dermis (Fig 2). These histologic findings supported the diagnosis of papular purpuric glove and socks syndrome.

Additional pertinent workup included parvovirus B19 serologies positive for IgG, but negative for IgM antibodies. Complete blood count with differential and comprehensive metabolic panel was unremarkable. Since the review of systems was not otherwise suggestive of another recent viral illness, no further viral workup was ordered. The patient did, however, have negative serologies for HIV and Hepatitis B/C 2 years before presentation.

The patient was continued on fluocinonide 0.05% ointment daily until her follow-up 16 days later, when there was a marked improvement in the pruritic eruption.

## Discussion

This case suggests that COVID-19 may be capable of inducing papular purpuric gloves and socks syndrome in a similar fashion as parvovirus B19 and other viral infections. It is worth noting that despite an apparent confounder in parvovirus serology, IgM tends to become positive within a few days to weeks and remain so for only 6 months or so, while IgG may persist for life, making a recent enough parvovirus infection unlikely.^3^^,^^4^ Moreover, the described exanthema has been observed after a number of viral infections.^5^ The histopathologic differential diagnosis may also include entities such as Gianotti-Crosti, hand-foot-mouth disease, erythema multiforme, and meningococcal sepsis,^6^ though these are less consistent with the distribution and overall clinical presentation of our patient's eruption. Understanding how COVID-19 may manifest on the skin may help clinicians and patients more effectively recognize and manage its sequelae.

## Conflicts of interest

None disclosed.
